# Rocking the Boat: The Decisive Roles of Rho Kinases During Oocyte, Blastocyst, and Stem Cell Development

**DOI:** 10.3389/fcell.2020.616762

**Published:** 2021-01-11

**Authors:** Islam M. Saadeldin, Hammed A. Tukur, Riyadh S. Aljumaah, Ramya A. Sindi

**Affiliations:** ^1^Department of Animal Production, College of Food and Agricultural Sciences, King Saud University, Riyadh, Saudi Arabia; ^2^Department of Comparative Medicine, King Faisal Specialist Hospital & Research Centre, Riyadh, Saudi Arabia; ^3^Department of Laboratory Medicine, Faculty of Applied Medical Sciences, Umm Al-Qura University, Makkah, Saudi Arabia

**Keywords:** rho kinase, oocyte, stem cells, differentiation, actin

## Abstract

The rho-associated coiled-coil-containing proteins (ROCKs or rho kinase) are effectors of the small rho-GTPase rhoA, which acts as a signaling molecule to regulate a variety of cellular processes, including cell proliferation, adhesion, polarity, cytokinesis, and survival. Owing to the multifunctionality of these kinases, an increasing number of studies focus on understanding the pleiotropic effects of the ROCK signaling pathway in the coordination and control of growth (proliferation, initiation, and progression), development (morphology and differentiation), and survival in many cell types. There is growing evidence that ROCKs actively phosphorylate several actin-binding proteins and intermediate filament proteins during oocyte cytokinesis, the preimplantation embryos as well as the stem cell development and differentiation. In this review, we focus on the participation of ROCK proteins in oocyte maturation, blastocyst formation, and stem cell development with a special focus on the selective targeting of ROCK isoforms, ROCK1, and ROCK2. The selective switching of cell fate through ROCK inhibition would provide a novel paradigm for *in vitro* oocyte maturation, experimental embryology, and clinical applications.

## Introduction

Rho-associated coiled-coil-containing protein serine/threonine kinases (ROCKs) are the most recognized and studied downstream effectors of the small GTPase RhoA, which regulates a plethora of cellular events, particularly actin-mediated cellular activities (Schwartz, 2004). With a primary role in the spatiotemporal organization of actin cytoskeletal-related cellular events, ROCK proteins act as the key that enables the interaction of actin cytoskeleton with molecular motors, that convert signals into forces essential for the regulation of cellular functions such as contraction, adhesion, migration, proliferation, and apoptosis (Riento and Ridley, 2003; Jaffe and Hall, 2005; Ohgushi et al., 2010). Ever since the discovery of the pleiotropic nature of ROCK, it has sparked the interest of various research and medical fields, including the pharmaceutical (Hahmann and Schroeter, 2009; Feng et al., 2015; Honjo and Tanihara, 2018; Knipe et al., 2018; Zhou, 2018), neuroregeneration (Wang et al., 2015), stem cell, and regenerative medicine (Krawetz et al., 2011; Li et al., 2015; Vernardis et al., 2017), cancer research (Street and Bryan, 2011; Kümper et al., 2016; Wei et al., 2016), and reproductive and developmental biology fields (Kawagishi et al., 2004; Laeno et al., 2013; Duan et al., 2014b; Miranda-Rodríguez et al., 2017; Tukur et al., 2020).

Structurally, ROCK consist of four domains: an amino (N-terminal) domain, a kinase domain, a central coiled-coil domain (known as the rho-binding domain, RBD), and a C-terminal domain. ROCK exists in two isoforms, ROCK1 and ROCK2, encoded by separate but closely related genes, *ROCK1* and *ROCK2*, respectively (Newell-Litwa et al., 2015; Rath et al., 2016). ROCK1 and ROCK2 are homologous and share about 64% amino acid sequence identity (Table 1). They differ in the activation mechanisms; both can be activated by binding of RhoGTP to the Rho binding domain (RBD). Meanwhile, ROCK1 can be activated through cleavage by caspase-3, while ROCK2 can be activated by granzyme B and caspase-2. Autophosphorylation of ROCK1 at Ser1333 and of ROCK2 at Ser1366 reflects the activation status of the kinases (Ishizaki et al., 1996; Nakagawa et al., 1996). Despite their structural similarity and similar action on the same downstream substrates, studies in murine models showed that ROCK1 and ROCK2 are expressed in different cells, tissue locations, and stages of development (Zhang et al., 2014; Zanin-Zhorov et al., 2016; Lu et al., 2020). They have different cellular localizations; ROCK1 possesses cytosolic localization and association with centrosomes (Chevrier et al., 2002), and the plasma membrane (Stroeken et al., 2006). In contrast, ROCK2 can be found in the cytoplasm and nucleus, associated with the centrosome, and co-localized with actin and vimentin filaments in different cell types (Leung et al., 1995, 1996; Sin et al., 1998; Katoh et al., 2001; Kawabata et al., 2004; Ma et al., 2006; Tanaka et al., 2006). Therefore, neither compensates for the loss of the other (Zhu et al., 2011). Specifically, ROCK2 is associated with blastocyst development in swine (Zhang et al., 2014).

**TABLE 1:** Comparison between ROCKs isoforms (ROCK1 and ROCK2).

	ROCK1	ROCK2	Sequence identity
Total amino acid length	1,354	1,388	Total 64%
N-terminal Kinase domain	76–338	92–354	90%
Coiled-coil region RBD domain	460–1,068	452–1,102	55%
	949–1,014	979–1,047	
C-terminal PH domain	1,120–1,318	1,152–1,345	65%

**PH, pleckstrin homology contains the cysteine-rich C1 domain (CRD). Coiled-coil region contains Rho binding domain (RBD).**

## General Mechanism of Rock Regulation and Function

**FIGURE 1:**
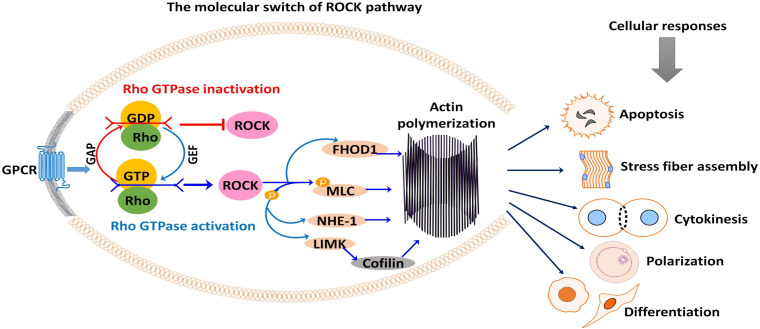
Rho-associated coiled-coil-containing protein serine/threonine kinase (ROCK) signaling activation and subsequent phosphorylation (activation/inhibition) of several downstream substrates are responsible for many cellular events. Signals from G-protein-coupled receptor (GPCR) stimulate the coupling of Rho with GEF or GAP, which switches ROCK on or off. As shown, ROCK downstream substrates are mostly involved in actin-mediated cellular activities such as apoptosis, stress fiber formation, cytokinesis, cell polarization, cell adhesion, and cell differentiation. GAP, GTPase-activating protein; GEF, guanine nucleotide exchange factor; GDP, guanosine diphosphate; GTP, guanosine triphosphate; FHOD1, formin homology 2 domain-containing 1; MLC, myosin light chain; NHE-1, Na^+^/H^+^ exchanger type 1; LIMK, LIM kinase.

For a detailed description of the general mechanism of ROCK, the reader is referred to the reviews of Amin et al. (2013) and Hartmann et al. (2015). Soluble hormones, cytokines, integrins, and growth factors bind to cell surface receptors to stimulate cellular responses. These stimuli trigger G-protein-coupled receptors to activate Rho-GTPases. This activation or deactivation depends on the coupling of Rho with the guanine nucleotide exchange factors (GTP and GDP), the molecular switch that regulates Rho-ROCK signaling (Dvorsky et al., 2004; Mott and Owen, 2015; Bagci et al., 2019). In the active form, Rho is bonded with GTP. Rho inactivation occurs when Rho-GTPase hydrolyzes GTP into GDP, switching Rho from GTP bounded (active) state to GDP bounded (inactive) form (Müller et al., 2020; Figure 1). Rho signaling is positively regulated through Rho guanine nucleotide exchange factors (RhoGEFs) that promote and catalyze the release of bound GDP for GTP (Cook et al., 2014). Meanwhile, Rho GTPase-activating proteins (RhoGAPs) are negatively regulating Rho signaling through stimulating the hydrolysis of Rho GTPases (Figure 1; Moon and Zheng, 2003). Additionally, Rho guanine nucleotide-dissociation inhibitors (Rho GDIs) can bind to and prevent the dissociation of GDP (Olofsson, 1999). If activated, Rho isoforms including RhoA, RhoB, and RhoC bind to and activate rho kinase (Sit and Manser, 2011).

Corroborating previous studies, the regulation of cell mechanics from stress fiber assembly, cell polarization, cell shape, and focal adhesion are mediated *via* RhoA GTPase–ROCK pathways. Recent findings by Lachowski et al. (2020) identified ROCK as a pivotal player in the transmission of signals through which the G-protein-coupled estrogen receptor (GPER) regulates actin polymerization rate and modulates cell polarization in fibroblasts.

Having identified the multifunctionality of ROCK, the contribution of the Rho/ROCK signaling was deeply explored, and several substrates linking ROCK to a variety of cellular activities were identified. After several works, rho kinase became a target molecule for cellular therapy, regenerative medicine, and stem cell research. A recent and noteworthy finding is the critical role of ROCK in Na^+^/H^+^ exchanger type 1 (NHE-1)-induced cell death. NHE-1 was previously known to regulate intermediate filament structure and cell shape. Recently, Wakabayashi et al. (2019) showed that the overexpression of NHE1 activated and sustained the ROCK pathway causing loss of human-induced pluripotent stem cells (iPSCs). This finding helped to elucidate the riddle behind the loss of iPSCs during cell passage after their dissociation.

## Downstream Targets of Rock

Activated ROCK phosphorylates various downstream substrates, including the myosin-binding subunit (MBS) of myosin phosphatase, myosin light chain (MLC), ezrin–radixin–moesin (ERM) proteins, LIM kinase, Na^+^/H^+^ exchanger type 1 (NHE-1), intermediate filaments vimentin (VIM) and glial fibrillary acidic protein (GFAP), formin homology 2 domain-containing 1 (FHOD1), and phosphatase and tensin homology (PTEN) (Schwartz, 2004; Schofield and Bernard, 2013; Hartmann et al., 2015). Through these substrates, ROCK mediates a wide range of cellular responses that involve the actin cytoskeleton, including cytoskeletal remodeling, actin–myosin contraction, actin filament stabilization, and microtubule dynamics (Figure 1). These cellular responses are entirely related to the cell cycle and meiotic maturation of oocytes, fate, and development of preimplantation embryos, and stem cell differentiation (Laeno et al., 2013; Duan et al., 2014b). Thus, ROCK has become a unique multifaceted protein of interest in oocyte, embryo, and stem cell research.

ROCK phosphorylates MLC and MBS to coordinate actinomyosin-mediated cellular contraction. Primarily, MLC phosphorylation causes cellular contraction. To further enhance this, MBS phosphorylation functionally inhibits MLC dephosphorylation by blocking myosin phosphatase activity, leading to an increase in MLC phosphorylation (Amano et al., 1996). This enables a conformational change that allows myosin II to assemble into bipolar filaments that bind to actin. Actin-activated ATPase activity increases, and eventually, actin stress fibers are formed. Focal adhesions stimulate actin–myosin interaction that induces tension, cellular contraction, stress fiber assembly, cell motility, proliferation, cytokinesis, cell adhesion, survival, and gene expression (Riento and Ridley, 2003). In particular, the ROCK pathway plays a pivotal role in cytoskeleton machinery required for cytokinesis, morphology, furrowing, and cell division; Rho-kinase stimulates polar relaxation, allowing the spindle to push and extend the cell sides permitting anaphase elongation. Moreover, Rho-kinase stimulates myosin II recruitment to the equatorial cortex, where it begins to contract in a broad zone permitting the contractile actin ring to assemble, and the cytokinetic furrow ingresses (Hickson et al., 2006). LIM kinase (LIMK) phosphorylates cofilin to prevent its binding with F-actin, inhibiting actin depolymerization and filament severing. Cofilin in turn promotes actin polymerization and stability. Recent works by Zhang et al. (2019) and Morales-Quinones et al. (2020) have clearly explained how LIMK implicates cytoskeletal organization and muscle contraction through the Rho pathway. The disruption of LIMK activities resulted in the loss of muscle contraction and vasoconstriction reducing cofilin phosphorylation and stiffening of arterial muscle (Morales-Quinones et al., 2020). Besides, the phosphorylation of ERM family proteins facilitates the linkage between actin and the plasma membrane. Furthermore, NHE-1 promotes both actin–membrane interactions and the function of the intermediate filament protein vimentin to regulate intermediate filament structure. The ROCK–cofilin–actin pathway is essential for meiotic development and cytokinesis during mouse oocyte maturation (Duan et al., 2014b). Results suggest that the ROCK/MLC/actomyosin, as well as ROCK/LIMK/cofilin pathways, regulate meiotic spindle migration and cytokinesis during bovine oocyte maturation (Lee et al., 2015). Besides, during cytokinesis, ROCK phosphorylates the intermediate filaments VIM and GFAP at the cleavage furrow, which is crucial for normal cell division. Slight distortion in the normal regulation of VIM and GFAP assembly/disassembly is associated with cytokinetic failure, aneuploidy, and bi-nucleation, which can result in cellular senescence (Matsuyama et al., 2013; de Pablo et al., 2019).

## Localization of Rho Kinase in Oocyte and Embryo

**FIGURE 2:**
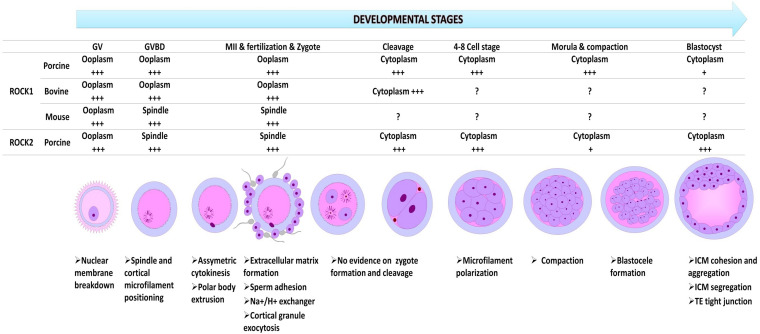
The relative spatial and temporal expression of ROCK1 and ROCK2 in oocytes and preimplantation embryos in different species, and their functions at every developmental stage. GV, germinal vesicle stage; Germinal vesicle breakdown at metaphase I (GVBD; MI); MII, metaphase 2; +++, richly expressed and predominantly distributed; +, very low expression; ?, no data available.

The distribution and function of ROCK in oocyte and embryo varies as development progresses suggesting a spatiotemporal role of ROCK at different stages of oocyte and embryo development (Figure 2). Northern blot analysis of Rho kinase protein, real-time PCR (rtPCR) analysis of Rho-kinase mRNA, and inhibition of ROCK has been employed to show the presence and involvement of Rho-kinase in the oocyte and embryo of rabbits (Stankova et al., 2014), mice (Duan et al., 2014a,b), cows (Hwang et al., 2013; Lee et al., 2015), felines (Arayatham et al., 2017), swine (Zhang et al., 2014), and camels (Tukur et al., 2020). In the bovine oocyte, ROCK was localized around the nucleus at the germinal vesicle (GV) stage but spread to the rest of the cytoplasm in later developmental stages (Lee et al., 2015). In the mouse oocyte, upon resumption of meiosis I, ROCK mRNA is localized around the spindles, colocalizing with cytoplasmic actin and mitochondria (Duan et al., 2014b), while it accumulated around the chromosomes at anaphase. At metaphase II, ROCK was again detected around the spindle. The localization pattern of ROCK was similar to that of cytoplasmic actin, which colocalizes with the centrosome in the cleavage furrow during mitosis (Riento and Ridley, 2003). Both ROCK 1 and ROCK 2 are very close in structure, function, and distribution; however, a distinction is observed in the amount and concentration of their mRNA and protein as development progresses (Zhang et al., 2014). A significant increase in ROCK1 mRNA and protein was clearly observed from the eight-cell and morula stages in swine, which then decreased significantly when blastocysts were formed (Zhang et al., 2014). An abundance of ROCK2 was observed at the blastocyst stage, indicating distinct roles for ROCK1 and ROCK2 at different stages of oocyte and embryo development. In porcine oocytes, ROCK1 accumulates in the ooplasm and after fertilization localizes in the cytoplasm from the two-cell stage to the blastocyst, while ROCK2 is found throughout the cytoplasm of the GV oocyte, and around the meiotic spindle at the MI and MII stage. In porcine preimplantation embryos, ROCK2 was detected in both the cytoplasm and the nucleus (Zhang et al., 2014).

## Rho Kinase and Oocyte Maturation

Oocyte maturation is a complex event that involves several crucial intercellular, intracellular, and molecular processes that lead to the generation of a mature and competent oocyte that can be fertilized by sperm and support early embryogenesis. Alterations in the normal choreography of this complex event have been associated with poorly developed oocytes, aneuploidy, miscarriage, infertility, and birth defects (Cimadomo et al., 2018; Bennabi et al., 2020). One unique feature of this event is asymmetric cytokinesis, without which successful oocyte maturation and fertilization cannot be achieved. Asymmetric cytokinesis depends on oocyte polarization, which is mediated by spindles that are assembled asymmetrically against the oocyte cortex, causing the expulsion of half of the chromosomes into tiny polar bodies. Spindle and cortical positioning and reorganization are crucial for polar body formation, as well as ensuring that the size of the polar body is appropriate, preventing loss of maternal cytoplasmic resources in the oocyte necessary for early embryo development (Barrett and Albertini, 2007; Clift and Schuh, 2013; Sun and Kim, 2013; Liu et al., 2018; Roeles and Tsiavaliaris, 2019). Through this process, the oocyte maintains a haploid chromosome number, as the other three genome copies are deposited into the polar bodies. These processes rely heavily on actin organization (Liu et al., 2018; Roeles and Tsiavaliaris, 2019; Santella et al., 2020).

Various factors that promote actin filament formation, migration, and assembly during the entire process of oocyte maturation have been exhaustively studied (Namgoong and Kim, 2016; Roeles and Tsiavaliaris, 2019). ROCK is among the kinases that regulate cytokinesis, control actin-binding proteins, and regulate Rho-mediated cellular activities, which include actin cytoskeletal organization, actin fiber formation, and actin dynamics in muscle cells and oocytes (Jaffe and Hall, 2005; Sit and Manser, 2011; Wang et al., 2017).

ROCK acts via the modulation of actin-binding proteins, including cofilin, profilin, FHOD1, tubulin, and FLNA during oocyte maturation (Wang et al., 2017). Particularly, ROCK inhibition decreased cofilin phosphorylation and caused defects in spindle migration, asymmetry, and polar body extrusion in mouse oocytes (Wang et al., 2017). This indicates a major role in cytoskeletal dynamics, spindle organization, and ultimately oocyte meiotic maturation, which is dependent on spindle positioning. During oocyte maturation (Figure 2), the germinal vesicle (GV), which is the nucleus of the oocyte must rupture, a phenomenon referred to as germinal vesicle breakdown (GVBD). The oocyte then re-initiates meiosis I and completes its development into a mature gamete. ROCK has been shown to be involved in the process of GVBD in bovine, mouse, porcine, and camel oocytes. Interestingly, this approach can be employed positively to control spontaneous meiotic resumption in immature oocyte *in vitro* maturation (IVM). However, prolonged inhibition of ROCK can have profound negative effects on oocyte and embryo development. In the camel, the prolonged inhibition resulted in either abnormal polar bodies or polar body failure (Tukur et al., 2020). Duan et al. (2014b) found that prolonged inhibition of ROCK activity during mouse oocyte maturation interfered with meiotic spindle migration and polar body extrusion. A similar phenotype was observed in bovine (Lee et al., 2015) and porcine (Suzuki et al., 2011) oocytes after ROCK inhibition. An improvement in fertilization and cleavage rate was observed, however, when ROCK was suppressed in vitrified feline oocytes (Arayatham et al., 2017). This could be attributed to the variation in the dose of ROCK inhibitor molecule that was used in the experiment or to the efficacy of specific dose on oocytes of different species. The dose of 10 μM of the ROCK inhibitor is commonly and widely reported.

Importantly, the ROCK pathway regulates the preparation of the oocyte to receive the sperm for fertilization on different levels (Rangel-Mata et al., 2007; Yodoi et al., 2009). Prostaglandin E2 signaling in cumulus cells negatively regulates Rho/ROCK signaling in cumulus cells to facilitate the fertilization process (Yodoi et al., 2009). ROCK/actomyosin pathway facilitates the surface accumulation of integrin and fibronectin fibril formation in the extracellular matrix of cumulus cells, which is a key event for interfering sperm penetration (Yodoi et al., 2009; Niringiyumukiza et al., 2018). Rho-kinase helps actin filament organization, cytoskeleton remodeling, and the action of Na^+^–H^+^ exchanger that is required for internal pH regulation and cortical granule exocytosis (Rangel-Mata et al., 2007). The ROCK–actomyosin pathway is therefore an essential requirement for oocyte fertilization by the sperm.

Many studies have focused on understanding the role of actin-binding proteins and have identified the regulatory pathways involved in oocyte development. It has been established that ROCK acts directly on MLC by direct phosphorylation (Lee et al., 2015; Liu et al., 2018), which increases cellular contraction, facilitating the interaction of myosin with F-actin. Further investigation revealed low levels of cortical actin, phosphorylated cofilin, and phosphorylated MLC when ROCK activity was interrupted (Lee et al., 2015). The LIMK is also directly phosphorylated by ROCK and regulates actin polymerization and microtubule dynamics via phosphorylation and inactivation of cofilin (Arayatham et al., 2017). In mice, LIMK was detected after GV breakdown, and it increased gradually from metaphase I to metaphase II, localizing to the microtubule organizing center (MTOC) of the spindle pole in MII oocytes.

## Rock and Blastocyst Development

There is increasing evidence that Rho-kinase is required for early mammalian embryonic development (Laeno et al., 2013) up to the blastocyst stage in mice (Kawagishi et al., 2004) and swine (Zhang et al., 2014). Rho-kinase mRNA has been detected in abundance at all stages of preimplantation and development (Kawagishi et al., 2004), promoting cleavage and blastocyst formation. Clayton et al. (1999) showed that inhibition of Rho-kinase with *Clostridium botulinum* C3-transferase disturbed intercellular flattening at compaction and prevented cytocortical microfilament polarization of eight-cell blastomeres. Additionally, treatment of porcine four-cell stage embryos and morula with Rho-kinase inhibitor Y-27632 resulted in defective morula compaction and cavitation (Son et al., 2010; Kwon et al., 2016). Similarly, the two-cell embryo treated with Y-27632 resulted in morula formation but inhibited the blastocoel formation and failed to develop blastocysts (Kawagishi et al., 2004; Kono et al., 2014). This was a result of inhibition of compaction and adhesion required for proper morphogenesis (Laeno et al., 2013). Moreover, Y-27632 reduced the rate of re-expansion of blastocysts when chemically collapsed by cytochalasin D suggesting that ROCK is involved in blastocyst formation (Kawagishi et al., 2004). Moreover, ROCK inhibition increased tight junction permeability of the trophectoderm through suppression of tight junction genes (such as CXADR, OCLN, TJP1, and CDH1) (Kwon et al., 2016) and reduced expression of trophectoderm-specific gene, CDX2 (Kono et al., 2014).

Surprisingly, inhibition of ROCK in cryopreserved vitrified (an ultra-rapid transition of liquid matter into an amorphous glass-like phase without ice crystal formation with the aid of liquid nitrogen) feline oocytes during IVM did not affect oocyte maturation and embryo developmental competence in terms of formation of the morula and blastocyst. Accordingly, the suppression of ROCK improves the revivability of feline (vitrified), human, and bovine blastocysts (Hochi et al., 2010; Huang et al., 2016; Arayatham et al., 2017). These reports suggest a positive effect of Rho-kinase inhibition on post-thawed embryos, rather than on fresh embryos. In fresh embryos, several reports (Kawagishi et al., 2004; Duan et al., 2014a) showed that the absence of ROCK significantly hinders oocyte and embryonic development.

## Roles of Rock in Embryonic Tissue Segregation and Stem Cell Differentiation

The blastocyst consists of the trophectoderm (TE), and the pluripotent inner cell mass (ICM) expands by the compaction of the ICM, which adopts an ovoidal shape. This process and morphology are crucial for normal embryogenesis. ROCK is required for cohesion of ICM cells, and the formation of segregated tissues called primitive endoderm (PrE) and epiblast (Epi), in the ICM of the mouse blastocyst (Laeno et al., 2013). ROCK inhibition with 20 μM Y-27632 caused dramatic changes in ICM cells and mouse embryonic stem cell line morphology and prevented cell aggregation and colony formation; they became flattened and spread as a monolayer on the culture dish, resulting in increased surface area of cultured cells, and this effect was mediated through inhibition of F-actin and microtubules (Laeno et al., 2013).

Rho-kinase, as an indispensable enzyme implicated in a plethora of cellular activities and functions, exhibited somewhat related yet distinct roles in different cell types. For instance, in stem cell differentiation, an investigation by Li et al. (2015) established in their study on bone marrow mesenchymal stem cells (BMSCs) that inhibition of ROCK with Y-27632 facilitated the differentiation of BMSCs into keratinocyte-like cells, and promoted the proliferation and survival of human primary keratinocytes. Likewise, suppression of ROCK with 20–50 μM Y-27632 promoted the differentiation of mouse embryonic stem cells into neurons (Kamishibahara et al., 2016) by activating the extracellular signal-regulated kinase signaling pathway. Similarly, the multipotency of rat sagittal suture mesenchymal stem cells showed a ROCK-dependent osteogenic differentiation under mechanical tension (Li et al., 2020). Furthermore, the ROCK pathway mediates the mechanical cues critical for neural stem cell differentiation (Kang et al., 2020).

Suppression of ROCK activity regulates the stem cells’ self-renewal and differentiation into the three germ layer derivatives. This suppression could be used as a tool for regular pluripotent stem cell culture and transportation/shipment and sharing cells between researchers to avoid geographic and logistic barriers (Kurosawa, 2012; Rivera et al., 2020; Ye et al., 2020). Recently, ROCK inhibitor fasudil showed improvement in the establishment of cloned embryo-derived embryonic stem cells through exerting an anti-apoptotic pathway (So et al., 2020a). It is, therefore, worthwhile to investigate how the modulation of Rho-kinase signaling can influence stem cell activities such as apoptosis (Joo et al., 2012; Li et al., 2015) and secretory activities (Zonderland et al., 2020). Particularly, mesenchymal stromal cell (MSC)-derived stanniocalcin-1 is critically regulated with ROCK signaling, owing to its importance in the anti-inflammatory, anti-apoptosis, and promoting angiogenesis associated with MSC therapy (Zonderland et al., 2020). Supplementing cell culture with ROCK inhibitor Y-27632 has proven to be a simple, efficient, and versatile approach for obtaining an abundance of embryonic stem cell-like cells at high purity suitable for use in regenerative medicine and therapeutics. Chapman et al. (2014) showed that long-term inhibition of Rho-kinase with Y-27632 improved the long-term proliferation of human keratinocytes, in addition to blocking and rescuing cells from aging. Joo et al. (2012) made a similar observation during the differentiation and proliferation of endothelial cells derived from an embryonic stem cell (ESC)-derived Flk1+ mesodermal precursor cells. Recently, Kim et al. (2020) found that ROCK inhibitor Y-27632 together with Matrigel improved the isolation, proliferation rate, and differentiation potential of urine-derived stem cells in humans. Furthermore, ROCK inhibitors have been used to promote proliferation, viability, and differentiation of mesenchymal stem cells (Baharvand et al., 2010; Mellott et al., 2014; Nakamura et al., 2014; Li et al., 2015).

Recent investigations in regenerative medicine and therapeutic studies have demonstrated the importance of rho kinase regulation in cell viability, proliferation, regeneration, and differentiation. The works of Yang et al. (2020) revealed that the addition of Y-27632 in the culture medium enhanced the viability of stem cells from human exfoliated deciduous teeth (SHED) and their differentiation into neuron-like cells. A corroborative result was reported by Baek et al. (2019) indicating the potential of rho kinase regulation.

Survival of cryopreserved human pluripotent stem cells (hPSCs) has been improved following the manipulation of Rho-kinase. Temporal inhibition of ROCK signaling represents a proven approach to prevent cell death and reinitiate cell proliferation after freezing or thawing pluripotent stem cells (So et al., 2020b). It has been suggested that ROCK inhibition increases the survival of hESCs because of continued and enhanced cell–cell interactions, reduced dissociation-induced apoptosis, and aided in the maintenance of pluripotent characteristics, and promoted cell propagation. Shi and Wei (2007) and Okumura et al. (2016) showed that cell death by apoptosis is mediated through the ROCK pathway; ROCK inhibition suppressed MLC phosphorylation, slowed cytokinesis, and prevented differentiation and cell death.

## Rock Inhibitors in Oocytes, Embryos, and Stem Cells

There is compelling evidence for the roles of ROCK signaling in oocyte meiotic and cytoplasmic maturation (Liu et al., 2018; Tukur et al., 2020), including those in embryo cleavage, blastocysts, and segregation of ICM (Kawagishi et al., 2004; Laeno et al., 2013). Moreover, ROCK signaling is pivotal in pluripotent and embryonic stem cells, where it is implicated in proliferation, differentiation, and survival (Zhang et al., 2011; Kamishibahara et al., 2016; Vernardis et al., 2017). In serum-free medium supplemented with the ROCK inhibitor, the survival of dissociated human embryonic stem cells was significantly improved. The inhibition of ROCK is not only useful in preventing and protecting hPSCs from apoptosis but also ensures that the phenotype of stem cells is maintained (Watanabe et al., 2007). This indicates that the ROCK inhibitors are a promising approach in stem cell research for the development of stem cell bioprocesses (Vernardis et al., 2017), and it may be worth exploring ROCK by extensively focusing on its modulation/inhibition in the control of cell growth, differentiation, fate, and development.

These studies established a rationale for the use of ROCK inhibitors as molecular tools to study the biological functions of ROCK and ROCK signaling in cellular events (Liao et al., 2007; Feng et al., 2015). Consequently, the biotechnology industry has developed several isoform-selective and non-selective ROCK inhibitors to determine the role of ROCK in a variety of cells and tissues (Liao et al., 2007; Defert and Boland, 2017; Saadeldin et al., 2020). Notably, ROCK inhibitors including fasudil and Y-27632 have been widely used in oocyte, embryo, and stem cell research (Laeno et al., 2013; Duan et al., 2014a; Defert and Boland, 2017; So et al., 2020b; Tukur et al., 2020). Interestingly, the selective efficacy of ROCK inhibitors is both dose and time dependent. For instance, Y27632, at a dose of 0.3 μM, blocks ROCK2, while ROCK1 appears sensitive and can be blocked at a lower dose of 0.22 μM (Liao et al., 2007). Paradoxically, SLx-2119 blocks ROCK1 at a concentration of 25 nM, while ROCK2 can be inhibited at lower concentrations of 5 nM (Shifrin et al., 2005). Moreover, the inhibitors H-1152P and ROKα inhibitor BF showed differential efficacy at different dosages (Liao et al., 2007). It was shown that Y27632 has time-dependent efficacy, especially when applied to dynamic meiotic oocytes; it exerted harmful effects when applied for 2, 4, 6, or 24 h during meiosis I, and beneficial effects when applied for 6 or 24 h after the first polar body extrusion (Tukur et al., 2020). Conversely, XD-4000 is a selective inhibitor for ROCK2. Several other inhibitors are under investigation for clinical purposes, although the most commonly used ROCK inhibitors in the fields of stem cell and developmental biology are fasudil and Y-27632, which act with similar efficiency (So et al., 2020b).

Interestingly, a photoactivatable small-molecule inhibitor was developed to be light-controlled for spatiotemporal control of ROCK activities in live embryos (Morckel et al., 2012). Researchers developed this caged Rockout (cRO) by attaching a 6-nitropiperonyloxymethyl (NPOM) moiety onto the indole nitrogen of Rockout (RO), which is degraded by exposure to UV light, thus restoring ROCK inhibitory activity. Adopting this cRO approach could facilitate fantastic discoveries in regenerative medicine, stem cell or tissue engineering, and organ culture, or other contexts that are affected by ROCK signaling (Zhang et al., 2009; Morckel et al., 2012). The weakness of ROCK inhibitors lies in their inability to distinguish between ROCK isoforms; hence, this approach may not be sufficient to understand the specific role of ROCK and the ROCK signaling pathways in different cells. A more reliable method will be a combination of this approach and genetic knockout models (Thumkeo et al., 2003; Rikitake et al., 2005).

## Conclusion and Future Directions

The spatial and temporal pleiotropic effects of ROCK isoforms control critical steps in oocyte meiosis, early embryonic stem cell lineage differentiation, and other pluripotent stem cell development; however, selective inhibitors of ROCK1 and ROCK2 isoforms are in the early stages of development. Selective ROCK inhibitors will be required to distinguish ROCK isoforms and to understand the critical roles of the ROCK pathway in cellular differentiation and elucidate their distinctive roles in oocyte, embryo, and pluripotent stem cell development. The most reliable method will likely be a combination of this approach and the use of gene knockout and/or knockdown animal models. Moreover, approaches such as cumulus-specific, oocyte-specific, and/or blastocyst-specific disruption of ROCK signaling molecules will be useful to address the physiological significance of Rho-ROCK signaling in the early steps of fertilization and embryonic development. ROCK targeting might be used for modulating the early developmental stages to control pregnancy such as novel targeted contraceptive drugs.

## Author Contributions

IS and HT conceptualized the study. IS, HT, RA, and RS wrote and edited the manuscript. All authors have read and agreed to the published version of the manuscript.

## Conflict of Interest

The authors declare that the research was conducted in the absence of any commercial or financial relationships that could be construed as a potential conflict of interest.
